# Supplementary material for: Single Cell Transcriptomic and Chromatin Profiles Suggest Layer Vb Is the Only Layer With Shared Excitatory Cell Types in the Medial and Lateral Entorhinal Cortex
Source: Front Neural Circuits. 2022 Jan 26;15:806154. doi: 10.3389/fncir.2021.806154 (PMC8826650; doi:10.3389/fncir.2021.806154)
Supplement: Supplementary file 14 [file Data_Sheet_1.ZIP › Exc_vs_Inh_Motifs/homerResults/motif9.similar.html]

motif9

## Information for motif9

C
T
G
A
C
A
G
T
C
T
A
G
A
G
C
T
C
G
T
A
G
T
A
C
G
A
T
C
G
C
T
A
G
T
A
C
G
T
C
A
  
Reverse Opposite:  

C
A
G
T
C
A
T
G
C
G
A
T
C
T
A
G
C
A
T
G
G
C
A
T
T
C
G
A
G
A
T
C
G
T
C
A
G
A
C
T
  

|  |  |
| --- | --- |
| p-value: | 1e-241 |
| log p-value: | -5.568e+02 |
| Information Content per bp: | 1.461 |
| Number of Target Sequences with motif | 27625.0 |
| Percentage of Target Sequences with motif | 65.49% |
| Number of Background Sequences with motif | 6258.1 |
| Percentage of Background Sequences with motif | 57.57% |
| Average Position of motif in Targets | 337.3 +/- 248.4bp |
| Average Position of motif in Background | 306.0 +/- 263.6bp |
| Strand Bias (log2 ratio + to - strand density) | 0.0 |
| Multiplicity (# of sites on avg that occur together) | 1.56 |
| Motif File: | file (matrix) reverse opposite |

### Similar de novo motifs found

|  |  |  |  |  |  |  |  |
| --- | --- | --- | --- | --- | --- | --- | --- |
| Rank | Match Score | Redundant Motif | P-value | log P-value | % of Targets | % of Background | Motif file |
| 1 | 0.664 | A C G T A C T G A C G T A C T G A C G T A C G T C G T A A G T C A C T G A C G T C G T A C G T A | 1e-172 | -396.928180 | 34.26% | 28.01% | motif file (matrix) |
